# Targeting of Both the c-Met and EGFR Pathways Results in Additive Inhibition of Lung Tumorigenesis in Transgenic Mice

**DOI:** 10.3390/cancers2042153

**Published:** 2010-12-22

**Authors:** Laura P. Stabile, Mary E. Rothstein, Phouthone Keohavong, Diana Lenzner, Stephanie R. Land, Autumn L. Gaither-Davis, K. Jin Kim, Naftali Kaminski, Jill M. Siegfried

**Affiliations:** 1 Department of Pharmacology and Chemical Biology, University of Pittsburgh, Pittsburgh, PA 15213, USA; E-Mails: las22@pitt.edu (L.P.S.); meb63@pitt.edu (M.E.R); gaitherdavisa@upmc.edu (A.L.G.D); 2 Department of Environmental and Occupational Health, University of Pittsburgh, Pittsburgh, PA 15213, USA; E-Mail: pho1@pitt.edu; 3 Department of Biostatistics, University of Pittsburgh, Pittsburgh, PA 15213, USA; E-Mails: del9@pitt.edu (D.L.); Land@nsabp.pitt.edu (S.R.L); 4 Galaxy Biotech, LLC, Sunnyvale, CA 94089, USA; E-Mail: jin.kim@galaxybiotech.com; 5 Department of Medicine, University of Pittsburgh, Pittsburgh, PA 15213, USA; E-Mail: nak38@pitt.edu; 6 Lung and Thoracic Malignancy Program, University of Pittsburgh, Pittsburgh, PA 15213, USA

**Keywords:** lung cancer, EGFR, HGF, c-Met

## Abstract

EGFR and c-Met are both overexpressed in lung cancer and initiate similar downstream signaling, which may be redundant. To determine how frequently ligands that initiate signaling of both pathways are found in lung cancer, we analyzed serum for hepatocyte growth factor (HGF), transforming growth factor-alpha, and amphiregulin (AREG) in lung cancer cases and tobacco-exposed controls. HGF and AREG were both significantly elevated in cases compared to controls, suggesting that both HGF/c-Met and AREG/EGFR pathways are frequently active. When both HGF and AREG are present *in vitro*, downstream signaling to MAPK and Akt in non-small cell lung cancer (NSCLC) cells can only be completely inhibited by targeting both pathways. To test if dual blockade of the pathways could better suppress lung tumorigenesis in an animal model than single blockade, mice transgenic for airway expression of human HGF were treated with inhibitors of both pathways alone and in combination after exposure to a tobacco carcinogen. Mean tumor number in the group using both the HGF neutralizing antibody L2G7 and the EGFR inhibitor gefitinib was significantly lower than with single agents. A higher tumor K-*ras* mutation rate was observed with L2G7 alone compared to controls, suggesting that agents targeting HGF may be less effective against mutated K-*ras* lung tumors. This was not observed with combination treatment. A small molecule c-Met inhibitor decreased formation of both K-*ras* wild-type and mutant tumors and showed additive anti-tumor effects when combined with gefitinib. Dual targeting of c-Met/EGFR may have clinical benefit for lung cancer.

## 1. Introduction

Non-small cell lung cancer (NSCLC) is typically diagnosed at late stages and does not respond well to current treatments, thus identification of improved therapies that target specific lung cancer growth pathways is essential. Targeting of receptor tyrosine kinase pathways using monoclonal antibodies and small-molecule inhibitors are promising approaches for NSCLC treatment. Many growth factor signaling pathways overlap and interact with each other, suggesting there is redundancy in how signaling is carried out. Strategies to interrupt receptor cross-signaling or to target more than one pathway may have increased effects on tumor inhibition.

Both c-Met and epidermal growth factor receptor (EGFR) are highly expressed in lung tumors and play important roles in the NSCLC progression [1,2]. The EGFR tyrosine kinase inhibitors (TKIs), gefitinib and erlotinib, have had limited clinical success in NSCLC patients. Certain patient subsets are particularly responsive to these drugs such as patients whose tumor harbors activating EGFR mutations within the EGFR TKI domain [3,4]. However, resistance eventually develops in patients after initial response due to a secondary EGFR mutation and/or c-Met amplification [5,6]. In the absence of an EGFR mutation, inherent resistance to EGFR TKIs may also occur because of redundant signaling from other tyrosine kinase receptors such as c-Met. Agents that target the c-Met pathway are currently being tested in clinical trials and have demonstrated success in preclinical lung cancer models [7]. However, maximizing effectiveness of targeted therapies may require combination approaches utilizing therapy against several molecular targets.

We have previously developed transgenic (TG) mice that overexpress the ligand for c-Met, human hepatocyte growth factor (HGF), only in the airways under control of the Clara cell secretory promoter. Furthermore, we have demonstrated that these mice have enhanced susceptibility to lung tumorigenesis, which is reversed upon administration of the anti-human HGF neutralizing antibody (NA), L2G7 [8,9]. These mice were shown to produce EGFR ligands in the lungs, suggesting the EGFR pathway is active. We have also previously demonstrated a reinforcing loop of c-Met-EGFR cross-activation initiated by HGF in lung cancer, that occurs via COX-2 activation and involves direct phosphorylation of c-Met by EGFR ligands [10,11]. Delayed phosphorylation of c-Met by EGFR does not require HGF and is an example of c-Met ligand-independent signaling. Because of the reported interactions between these two overlapping pathways by our group and others [12], we looked for evidence of pathway activation for both c-Met and EGFR in NSCLC patients, and for an increased anti-tumor effect in the HGF TG murine model from dual targeting of both HGF/c-Met and EGFR pathways. We demonstrate that reducing two intersecting signaling components may improve therapeutic efficacy for NSCLC. 

## 2. Results and Discussion

### 2.1. Serum Analysis for Circulating c-Met and EGFR Ligands in Lung Cancer Cases and Controls

We have previously reported that HGF is a negative prognostic factor for lung cancer and that HGF protein expression is increased in lung tumors compared to normal lung [15]. We and others have also shown there is cross-talk between the c-Met and EGFR signaling pathways in lung cancer [10,11,12]. To determine extent of utilization of both pathways, we measured circulating levels of both c-Met and EGFR ligands in lung cancer patients compared to controls. Serum levels of HGF, transforming growth factor-alpha (TGFα), and AREG were examined in 71 subjects, 43 lung cancer cases and 28 smoking controls without lung cancer. Cases and controls were comparable in all subject characteristics except for age. Cases had a higher mean age than controls and a greater range of ages (Table 1). Age was not related to serum level of ligands, and was adjusted for in all analyses. Lung cancer cases showed elevated expression of both HGF and AREG ligands relative to controls (p < 0.001 for both) (Table 2). The median HGF level was 1657.50 pg/mL (range 1020.00–4855.00 pg/mL) in the lung cancer case group compared to 1025.72 pg/mL (range 592.08–4029.94 pg/mL) in the control group. The median AREG level was 25.19 pg/mL (range 6.89–37.10 pg/mL) in cases and 1.33 pg/mL (range 0.00–204.30 pg/mL) in controls. Using dichotomized TGFα expression, (TGFα = 0 / TGFα > 0), there was no significant difference in TGFα expression between cases and controls (TGFα = 0, 32 cases and 18 controls, p = 0.429). Kappa’s coefficient of 0.38 (H_0_:Κ = 0, p = 0.002) suggested fair to moderate agreement in “high/low” expression levels of HGF and AREG in all subjects.

We next determined whether factors such as age, smoking or presence of COPD (Chronic Obstructive Pulmonary Disease) were responsible for differences observed in HGF, TGFα or AREG serum levels between cases and controls (Table 3). When modeling by logistic regression the probability of high marker expression for each of the three ligands, 11 observations were excluded due to missing values of the explanatory variables (six unknown COPD status, three with a smoking status of unknown, never, or smoker NOS, and two with both unknown COPD status and unknown smoking status). Among the remaining 60 subjects, cases still had a higher probability of high HGF and AREG serum levels than controls (p < 0.001, OR 23.25 and p < 0.001, OR 43.05, respectively) while TGFα was not significant (Table 3). Nineteen of 32 (59%) cases showed levels of both HGF and AREG above the median for all subjects (cases and controls combined), compared to 0 of 28 controls (p = 0.009)*.* Among all subjects, 25 of 43 (58%) cases showed levels of both HGF and AREG above the medians, whereas none of the 28 controls had both HGF and AREG above the medians (p = 0.002). Based on our models, age, sex, smoking status and COPD were not factors in HGF or AREG serum levels. Subjects with COPD had a greater probability of high TGFα score than those without COPD (p = 0.016, OR = 9.19), suggesting that TGFα may be related to lung function. Older age was also associated with lower TGFα levels. Among cases, histology, age, sex, smoking status and COPD status were not significant predictors of high AREG, high HGF or high TGFα serum levels. Patients with stage III/IV disease had a greater probability of high HGF than patients with stage I/II disease (p = 0.039, OR = 16.6), however this was not observed for AREG or TGFα.

**Table 1:** Subject characteristics.

		Case		Control		Total		p-value*
		N	(%)	N	(%)	N	(%)	
**TOTAL Number of subjects**		43		28		71		
**SEX**								
	male	23	(53%)	13	(46%)	36	(51%)	0.631
	female	20	(47%)	15	(54%)	35	(49%)	
**SMOKING STATUS**								
	current smoker	20	(47%)	14	(50%)	34	(48%)	1.00
	ex-smoker	18	(42%)	14	(50%)	32	(45%)	
	never smoker	1	(2%)	0	(0%)	1	(1%)	
	smoker, NOS	1	(2%)	0	(0%)	1	(1%)	
	unknown	3	(7%)	0	(0%)	3	(4%)	
**COPD/EMPHYSEMA**								
	yes	21	(49%)	14	(50%)	35	(49%)	0.456
	no	14	(33%)	14	(50%)	28	(39%)	
	unknown	8	(19%)	0	(0%)	8	(11%)	
**HISTOLOGY**								
	squamous	18	(42%)	NA		18	(25%)	
	adenocarcinoma, adeno-squamous	17	(40%)			17	(24%)	
	NSCLC, small cell, giant cell	8	(19%)			8	(11%)	
						NA = 28	(39%)	
**STAGE**								
	I	17	(40%)	NA		17	(24%)	
	II	11	(26%)			11	(15%)	
	III-IV	14	(33%)			14	(20%)	
	unknown	1	(2%)			1	(1%)	
						NA = 28	(39%)	
**AGE**								
	mean	66.81		61.75		64.82		0.036
	std.dev	11.13		7.05		9.99		
	median	67.00				65.00		
	(min, max)	(38.0, 92.0)		(51.0, 71.0)		(38.0, 92.0)		

*All p-values are from Fisher’s Exact Tests except for the one for age, which is from a t-test.

**Table 2:** HGF and AREG serum levels in cases and controls.

**HGF**	**Group**	**N**	**Median (Range)**	**p-Value**
	Case	43	1657.50 (1020.00–4855.00)	<0.001
	Control	28	1025.72 (592.08–4029.94)	
**AREG**	**Group**	**N**	**Median (Range)**	**p-Value**
	Case	43	25.19 (6.89–37.10)	<0.001
	Control	28	1.33 (0.00–204.30)	

**Table 3:** Association of patient characteristics with HGF, TGFα and AREG serum level.

**HGF: **Probability of High HGF (HGF ≥ 1510 pg/mL)							
**Parameter**		**DF**	**Wald**			**Wald 95%**	
			**Chi-Square**	**Pr > ChiSq**	**Odds Ratio**	**Confidence Limits**	
Intercept		1	2.400	0.1219			
CASE/CTRL	Case	1	16.000	**<0.0001**	23.250	4.976	108.630
AGE		1	1.950	0.163	1.061	0.976	1.152
SEX	Female	1	1.960	0.161	0.357	0.085	1.508
SMOKING STATUS	Current Smoker	1	2.770	0.096	3.825	0.788	18.558
COPD	COPD	1	0.150	0.703	1.352	2.87	6.37
**AREG:** Probability of High AREG (AREG ≥ 23.8 pg/mL)							
**Parameter**		**DF**	**Wald**			**Wald 95%**	
			**Chi-Square**	**Pr > ChiSq**	**Odds Ratio**	**Confidence Limits**	
Intercept		1	0.074	0.785			
CASE/CTRL	Case	1	20.802	**<0.0001**	43.048	8.547	216.83
AGE		1	0.155	0.694	0.982	0.898	1.074
SEX	Female	1	0.192	0.662	0.722	0.168	3.102
SMOKING STATUS	Current Smoker	1	0.660	0.417	1.986	0.380	10.393
COPD	COPD	1	0.047	0.829	1.194	0.238	5.983
**TGFα: **Probability of High TGFα (TGFα > 0 pg/mL)							
**Parameter**		**DF**	**Wald**			**Wald 95%**	
			**Chi-Square**	**Pr > ChiSq**	**Odds Ratio**	**Confidence Limits**	
Intercept		1	4.602	0.032			
CASE/CTRL	Case	1	1.065	0.302	0.510	0.142	1.833
AGE		1	6.001	**0.014**	0.889	0.809	0.977
SEX	Female	1	2.804	0.094	0.325	0.087	1.211
SMOKING STATUS	Current Smoker	1	0.091	0.763	0.806	0.199	3.267
COPD	COPD	1	5.803	**0.016**	9.193	1.512	55.891

We next examined whether there was a significant relationship between the number of elevated circulating ligands and case status in all subjects using the Jonckheere Terpstra trend test. There was a significant relationship between the number of circulating ligand levels of HGF and AREG and case status (p < 0.001). Twenty-one controls (75%) had no elevated ligand levels compared to three cases (7%). No controls had elevated ligand levels for both the c-Met and EGFR receptors whereas 25 cases (58%) had elevated ligand levels for both receptors. This suggests that lung cancer patients can easily be identified who have evidence of both c-Met and EGFR pathway activation because of elevated circulating ligand levels for both receptors.

### 2.2. In the Presence of both c-Met and EGFR Ligands, Inhibitors of Both Pathways Are Necessary for Full Signaling Inhibition

**Figure 1:**
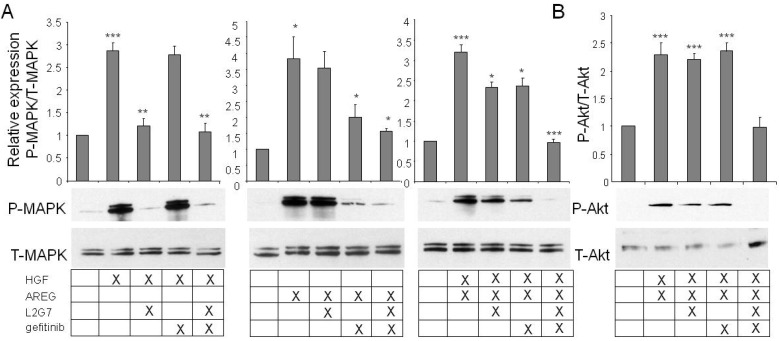
201T lung cancer cells were serum deprived for 48 h followed by treatment with 10 ng/mL HGF, 10 ng/mL AREG for 10 min or pretreatment with 300 ng/mL L2G7, 20 μM gefitinib or a combination of the two inhibitors for 2 h. Immunoblots were performed for (**A**) P-MAPK and T-MAPK and (**B**) P-Akt, and T-Akt. Representative immunoblots are shown for each treatment set. Immunoreactive bands from 3 independent experiments were quantitated and expressed as the ratio of P-MAPK or P-Akt to T-MAPK or T-Akt relative to control for each blot. *p < 0.05, **p < 0.005, ***p < 0.0005. Comparisons were controls *vs.* ligand and ligand treatment *vs.* inhibitors using Student’s t-test.

Signaling through c-Met may compensate for EGFR inhibition, and *vice versa*. We tested the effect of specific inhibitors of the c-Met and EGFR pathways alone and in combination in the presence of ligands for each pathway in NSCLC cells (Figure 1). 201T cells were selected for use because they express moderate levels of c-Met and EGFR, have no c-Met or EGFR amplification and no c-Met, EGFR or K-*ras* mutation, and are thus similar to the majority of NSCLC [10,16]. In 201T cells, HGF alone stimulated a 2.7-fold increase in P-MAPK expression and only L2G7 could inhibit the HGF-induced P-MAPK signaling, while gefitinib could not. In the presence of AREG (3.3-fold stimulation of P-MAPK), only gefitinib could inhibit AREG-induced P-MAPK signaling, while L2G7 could not, thus demonstrating the specificity of each inhibitor. When ligands of both pathways are present, inhibitors of both pathways are necessary to achieve full signaling inhibition, showing that c-Met and EGFR signaling can substitute for each other (Figure 1A). Partial inhibition was observed for each inhibitor alone in the presence of both ligands. Similar results were observed with HGF and TGFα in place of AREG (data not shown). In two additional NSCLC cell lines, 784T and H23 cells (both EGFR and c-Met wild-type), the same effects were observed (data not shown).

To provide additional evidence for redundancy of c-Met and EGFR signaling, we also analyzed another downstream signaling molecule activated by HGF and AREG, P-Akt. We observed similar results when examining P-Akt (Figure 1B) as with P-MAPK; HGF plus AREG induced a 2.3-fold increase in P-Akt expression and inhibitors of both pathways were necessary to achieve complete inhibition of P-Akt.

### 2.3. Inhibition of Tumorigenesis is Maximal with Combination Treatment

We previously used the HGF NA, L2G7, to block c-Met action in animals [9]. L2G7 is specific to human HGF and cannot block murine HGF, which is produced in human lung xenografts by the murine stromal cells in a paracrine manner. The HGF TG animal model we developed, which is driven by human HGF expressed in the lungs, is essential to demonstrate the action of L2G7 *in vivo* against lung cancer. EGFR is expressed at moderate levels in the lungs in this animal model which responds to EGFR TKIs and the EGFR ligands, TGFα, EGF and AREG are all expressed in lung tissue and bronchioalveolar lavage fluid isolated from these animals (data not shown). EGFR mutations are not found in mice when tumors are induced by a tobacco carcinogen. The observations we made previously demonstrating a reinforcing loop of c-Met-EGFR activation through ligand-independent c-Met phosphorylation suggests that dual blockade of these pathway elements could further suppress lung tumor development in an HGF-rich environment [10]. We tested whether addition of EGFR blockade might further suppress lung tumorigenesis in an HGF-rich environment by exposing HGF TG mice to the tobacco carcinogen, NNK, followed by treatment with L2G7, isotype-matched IgG, or gefitinib, alone and in combination.

**Figure 2:**
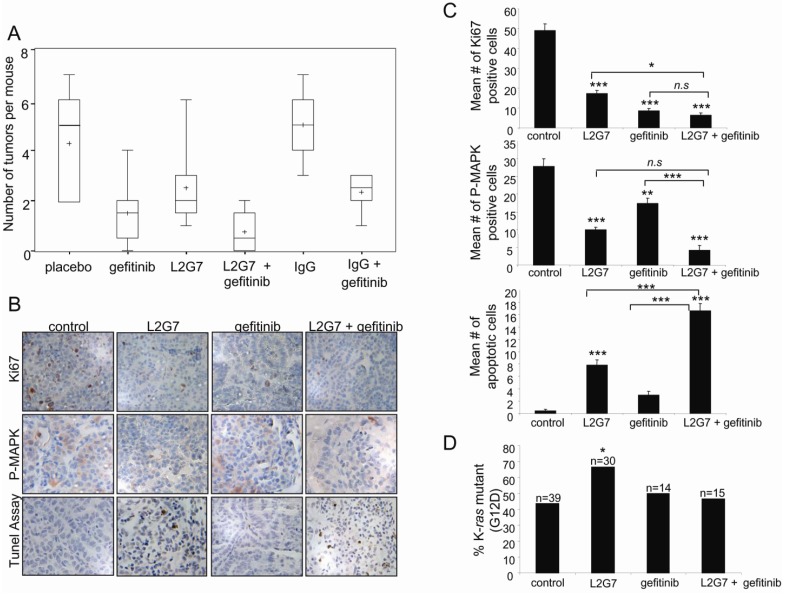
Tumor formation following NNK exposure in HGF transgenic mice treated with placebo, L2G7, gefitinib, or control IgG isotype-matched control antibody. (**A**) Boxplot of the number of tumors per mouse by treatment group. Line represents median and + represents the mean for each group. (**B**) Representative tumor sections from placebo control, L2G7, gefitinib and L2G7 + gefitinib treated animals showing immunohistochemical staining for Ki67, P-MAPK and apoptotic cells. (**C**) Quantitation for each marker was performed and results are presented as the mean ± SE number of positive cells for each marker from 5-high powered fields per experimental treatment. * P < 0.05; ** P < 0.01; *** P < 0.001 compared to control unless indicated, ANOVA. (**D**) Percentage of individual tumors containing a K-*ras* mutation. Control represents tumors isolated from placebo-treated and IgG-treated animals. Results analyzed using Fisher’s Exact Test.

A boxplot representing the mean and median number of tumors per animal in each treatment group is shown in Figure 2A. Mice were assigned to one of six treatment groups (n = the number of animals per group): placebo control (n = 11), L2G7 (n = 18), gefitinib (n = 19), L2G7 + gefitinib (n = 22), IgG control (n = 6), or gefitinib + IgG control (n = 6). We have previously shown that there is no difference in tumor formation between IgG- and placebo-treated animals thus, IgG and IgG + gefitinib treatment groups were performed in a small subset of animals to ensure that the effect of gefitinib was not altered in the presence of an antibody. The mean number of tumors per animal was significantly lower in the L2G7 (mean = 2.5; range = 1–6; p = 0.010), gefitinib (mean = 1.37; range = 0–4; p < 0.001) and L2G7/gefitinib combination groups (mean = 0.82; range = 0–3; p < 0.001) compared to placebo (mean = 4.27; range = 2–7). Likewise, the mean number of tumors per animal was significantly lower in the L2G7 (p = 0.003), gefitinib (p < 0.001), and L2G7/gefitinib combination (p < 0.001) groups compared to IgG control-treated group (mean = 5.00; range = 3–7). Mean number of tumors from the L2G7/gefitinib combination-treated group was significantly lower compared to that in the L2G7 alone-treated group (p < 0.001) and the gefitinib + IgG-treated group (mean = 2.30; range = 1–3; p = 0.003). The combined effect of gefitinib alone and L2G7 alone (−1.14–0.54 = −1.65) is equal to effect of the L2G7/gefitinib combination group (−1.65), showing the effect is additive.

IgG control antibody had no effect compared to placebo-treated animals (p = 0.501) while IgG + gefitinib has no effect *vs.* gefitinib alone (p = 0.107). When combining the placebo control and IgG control-treated groups into one control group (all controls) and the gefitinib and gefitinib + IgG into one gefitinib group (all gefitinib) the data remained significant: all controls *vs.* L2G7 (p = 0.002); all controls *vs.* all gefitinib (p < 0.001); all controls *vs.* L2G7/gefitinib combination (p < 0.001); L2G7 *vs.* combination (p < 0.001); and all gefitinib *vs.* combination (p = 0.018). 73%, 21% and 33% of the animals in the combination, L2G7 and all gefitinib groups, respectively, developed only one or no detectable tumors while all animals developed two or more tumors in the all control group. There was not a statistically significant difference in tumor sizes between treatment groups (p = 0.36). 

### 2.4. Maximum Inhibition of Indicators of Lung Tumor Growth and Survival Is Observed with Dual Therapy

Recently it has been shown that the c-Met and EGFR signaling networks overlap extensively in survival and proliferation parameters, thus inhibitors of these pathways alone may be clinically ineffective [17]. We examined the effect on these downstream events by examining expression of P-MAPK, Ki67 and apoptotic cells in tumors induced by NNK as indicators of relative tyrosine kinase signaling, proliferation, and cell survival (Figure 2 B, immunohistochemistry pictures; Figure 2C, quantitation). The mean number of Ki67 and P-MAPK positive cells, respectively, was decreased 65% and 64% by L2G7, 82% and 37% by gefitinib and 86% and 85% by combination treatment. Ki67 labeling was significantly lower in the combination-treated group compared to L2G7 alone but not gefitinib alone while P-MAPK expression was inhibited more by L2G7 than gefitinib. Apoptosis was significantly higher in the combination treated group (17-fold) compared to each single agent (7.9- and 3.5-fold for L2G7 and gefitinib, respectively). Inhibition of MAPK alone appears insufficient for maximal blockade of tumor growth, while apoptosis appears to be a good indicator of the increased effect of dual inhibition.

### 2.5. K-ras Mutant Tumors Are Partially Resistant to HGF Blockade

We have previously shown that the HGF transgene promotes both K-*ras* mutant and wild-type tumors and that L2G7 treatment alone preferentially inhibits the formation of K-*ras* wild-type tumors [9]. To determine how K-*ras* mutation affects sensitivity to dual targeted therapy, laser capture microdissection was used to isolate tumors from tissue sections for K-*ras* mutation analysis in codons 12 and 13 where most ras activating mutations are located. Thirty-nine tumors were isolated from the placebo and IgG-treated groups, 30 from the L2G7-treated group, 14 from gefitinib-treated and 15 from the L2G7/gefitinib combination group. The latter two groups yielded fewer tumors per animal for analysis. Some tumors had been depleted from previous assays thus the entire dataset could not be analyzed. Fig. 2D shows that with L2G7 treatment, which produces about a 50% inhibition of tumorigenesis, tumors that did form were more likely to be K-*ras* mutant (p = 0.041, Fisher’s exact test), confirming our previous report [9]. K-*ras* mutations were found in 66.7% (20/30) of tumors from L2G7-treated, 50.0% (7/14) of tumors from gefitinib-treated, and 46.7% (7/15) from combination-treated animals. The placebo-treated and IgG-treated groups had identical frequencies of mutation and were combined into one all control group with a 43.6% (17/39) K-*ras* mutant frequency. All mutations found were G12D in codon 12.

### 2.6. Confirmation of Dual Targeting

**Figure 3:**
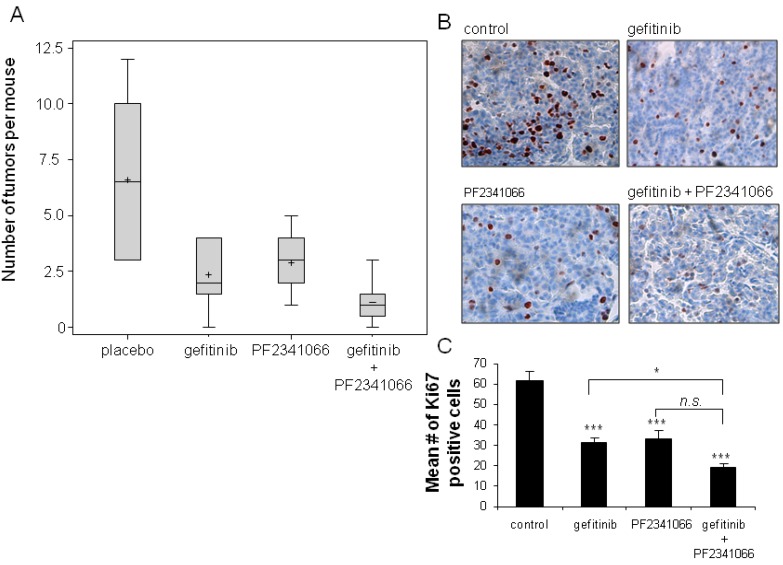
Tumor formation following NNK exposure in HGF transgenic mice treated with placebo, PF2341066, gefitinib, or PF2341066 in combination with gefitinib. (**A**) Boxplot of the number of tumors per mouse by treatment group. Line represents median and + represents the mean for each group. (**B**) Representative tumor sections showing immunohistochemical staining for Ki67. (**C**) Quantitation for Ki67 was performed and results are presented as the mean ± SE number of positive cells for each marker from 5-high powered fields per experimental treatment. * P < 0.05; *** P < 0.001 compared to control unless indicated, ANOVA. n.s. = non-significant.

Comparable results were obtained with dual targeting of EGFR and the c-Met pathways *in vivo* in an additional NNK tumorigenesis experiment using gefitinib and PF2341066, a c-Met tyrosine kinase inhibitor, alone and in combination. Gefitinib (n = 8 mice) resulted in a mean of 2.38 tumors/mouse compared to 6.6 tumors/mouse in placebo control (n = 10 mice, p < 0.001). PF2341066 (n = 9 mice) was also effective compared to control, yielding 2.9 tumors/mouse (p < 0.001). The combination of gefitinib and PF2341066 yielded a mean of 1.13 tumors/mouse (n = 8 mice, p < 0.001 combination *vs*. placebo; p = 0.015 combination *vs*. PF2341066 alone; p = 0.065 combination *vs.* gefitinib alone). A boxplot representing the mean and median number of tumors per animal in each treatment group is shown in Figure 3A. The effects of PF2341066 and gefitinib act additively with the effects of each treatment alone equal to the effect of the combination.

We also examined Ki67 as a marker of cellular proliferation in the tumors from these treatment groups. Figure 3B, C show representative images from the different treatment groups and quantitation, respectively. As with the results with L2G7, the tumors treated with the c-Met inhibitor in combination with gefitinib showed the lowest number of Ki67 positive cells. The mean number of Ki67 positive cells was decreased 45.3% by PF2341066, 48.2% by gefitinib and 68.1% by the combination. K-*ras* mutation status was also determined from the tumors generated in this experiment. Of a total of 22 tumors that were isolated from the PF2341066 treatment group, 11 of 22 were K-*ras* mutant (50%) similar to the percentage of K-*ras* mutant positive tumors for control and gefitinib alone treatment groups (p = 1.0; PF2341066 compared to control, Fisher’s exact test). Three of nine tumors (33.3%) were K-*ras* mutant from the combination PF2341066 plus gefitinib treatment group (p = 0.71, combination compared to control). In contrast to targeting the HGF ligand, targeting the receptor for HGF blocked both K-*ras* wild-type and K-*ras* mutant lung tumors.

## 3. Experimental Section

### 3.1. Reagents

L2G7 anti-human HGF monoclonal antibody and IgG isotype matched antibody control were obtained from Galaxy Biotech (Mt. View, CA). Nitrosoamine 4-(methylnitrosoamino)-1-(3-pyridyl)-1-butanone (NNK) was from Toronto Research Chemicals (North York, ON, Canada). Gefitinib was purchased from ChemiTek (Indianapolis, IN). 201T and 784T cells were established as described previously [13]. H23 cells were purchased from ATCC. All cell lines have been authenticated by genotyping. PF2341066 was provided by Pfizer (San Diego, CA).

### 3.2. Western Analysis

Cells (75% confluent) were serum-deprived for 48 h followed by addition of recombinant human HGF and/or AREG (10 ng/mL), TGFα (10 ng/mL), L2G7 (300 ng/mL), and/or gefitinib (20 μM) to the cells as indicated in the figure legend. Gefitinib was added for 2 h prior to ligand stimulation. L2G7 was incubated in the presence of ligands for 2 h prior to addition to the cells. Protein was extracted 10 min after ligand addition and quantified as described [9]. Western analyses for phospho-p44/p42 mitogen-activated protein kinase (P-MAPK), total-p44/p42 MAPK (T-MAPK), P-Akt and T-Akt were performed as described previously [9]. Immunoreactive bands were quantitated by densitometry and ImageQuant analysis.

### 3.3. Mouse Model

All mice used for experiments were human HGF transgenic (FVB/N strain), permissive in the airways under control of the Clara cell secretory promoter, and heterozygous for the transgene with high copy number as described previously [8]. The mice show enhanced lung tumorigenesis compared to their wild-type littermates [8]. Wild-type mice produce only murine HGF which is not inhibited by the human-specific L2G7 antibody, currently in clinical trials. A human HGF transgenic model is needed to demonstrate its anti-tumor effects in mice. Breeding and identification of transgenic mice were as described previously [8]. Mice were given a total of eight i.p. injections of 3 mg NNK (15 μg/μL) over 4 weeks. Beginning at week 3, treatment with L2G7 (100 μg, i.p), IgG control (100 μg, i.p.), and/or gefitinib (150 mg/kg, p.o.) was initiated and continued through week 15. PF2341066 (1 mg, p.o.) was administered 5 X/week for 3 week intervals followed by one week of no treatment, beginning at week 4, alone or with gefitinib (150 mg/kg, p.o.). At week 15, animals were sacrificed, lungs were processed and tumors analyzed as described [9]. Animal care was in strict compliance with the institutional guidelines established by the University of Pittsburgh.

### 3.4. Immunohistochemistry (IHC)

Lungs were fixed in 10% buffered formalin. Lung samples were paraffin embedded, sliced and mounted on slides. Slides were stained and quantitated for P-MAPK and Ki67 as described previously [9]. The number of apoptotic cells was determined using the ApopTag® Peroxidase In Situ Apoptosis Detection Kit (Intergen Company, Purchase, NY) as described previously [9]. 

### 3.5. Laser Capture Microdissection of Tumors and K-ras Mutation Analysis

Laser capture microdissection, DNA isolation and K-*ras* mutation analysis were performed as described previously [9]. Nested PCR was performed to amplify K-*ras* exon 1 (codon 12/13) followed by denaturing gel electrophoresis to separate mutant from wild-type alleles. Direct sequencing was performed to confirm the mutations.

### 3.6. Ligand Analysis in Human Serum

Serum from 71 individuals, with or without lung cancer, was collected under an approved protocol by the University of Pittsburgh Institutional Review Board. Controls were participants in the Pittsburgh Lung Screening Study (PLuSS), who received pulmonary function testing and a blood draw at study entry [14]. Control subjects had a baseline CT scan that was negative for lung cancer and they remained cancer-free during a minimum 3 year follow-up. Cases were identified through the thoracic oncology service at the University of Pittsburgh. Pulmonary function status of cases was ascertained from medical records. Cases received a biopsy-proven lung cancer diagnosis; blood was collected at diagnosis. Bloods were processed under the same protocol to obtain serum, which was analyzed in duplicate for HGF, TGFα, and AREG circulating levels using commercially available ELISA kits according to the manufacturers’ instructions. TGFα and HGF Quantikine ELISA kits and AREG DuoSet ELISA kit were from R&D Systems (Minneapolis, MN). Subject characteristics are provided in Table 1.

### 3.7. Statistical Analysis

Differences in expression of ligands between cases and controls were examined by Wilcoxon-Mann-Whitney tests for HGF and AR and Fisher’s exact test for TGFα which was dichotomized into TGFα = 0 and TGFα > 0. TGFα was dichotomized because most subjects (50 out of 71 subjects) had no detectable TGFα expression in serum. 

Logistic regression was used to model the probability of high expression of HGF, TGFα or AREG as a function of case status (case *vs.* control), age, sex, smoking status (current smoker *vs.* ex-smoker) and chronic obstructive pulmonary disease (COPD) status. Values of HGF and AREG expression were dichotomized into high and low expression at the median of all subjects (1,510 pg/mL for HGF and 23.8 pg/mL for AREG). The Jonckheere-Terpstra test for trend was used to examine the relationship between the number of circulating ligands and case/control status for all samples.

In the mouse model, Poisson regression was used to estimate the mean number of tumors per mouse in each treatment group and linear contrasts were used to determine which groups differed significantly from one another. To examine the differences in tumor sizes between treatment groups, we used a linear mixed effects regression model. 

For *in vitro* studies, Student’s t-test was used. For immunohistochemical quantitation, ANOVA was used. All statistical tests were two-sided with the threshold for statistical significance defined as P < 0.05. 

## 4. Conclusions

Using a transgenic mouse that overexpresses human HGF in the airways, we have previously demonstrated that the increased susceptibility of this mouse strain to lung cancer induction by a tobacco carcinogen can be reversed by a neutralizing antibody to human HGF. Furthermore, we have shown that tumors resistant to the HGF NA exhibit a higher rate of K-*ras* mutation than observed in the mice treated with an isotype matched control antibody suggesting that the ability to target the HGF ligand therapeutically may be less effective in lung tumors with an activating K-*ras* mutation. We now show evidence from patient serum that circulating ligands for the c-Met pathway as well as the EGFR pathway are frequently high in subjects with lung cancer compared to controls, that signaling via these two pathways is redundant in lung cancer cells *in vitro*, and that targeting these two pathways simultaneously in our carcinogenesis animal model results in increased anti-tumor effects. 

Our results show that signaling through c-Met results in activation of many of the same pathways activated by EGFR in NSCLC cells, including P-MAPK and P-AKT. The c-Met pathway can substitute for the EGFR pathway in NSCLC cells to stimulate cell signaling in the setting of inhibition of EGFR and *vice versa*. It has been demonstrated in lung cancer that EGFR and c-Met are linked via ERBB3 and this has been implicated in clinical resistance to EGFR TKIs [6]. Furthermore, EGFR ligands can activate c-Met independently of HGF in lung cancer cells, allowing for signaling via HGF-dependent and HGF-independent mechanisms both of which may contribute to c-Met mediated effects [11]. We show here that full inhibition of both the P-MAPK and P-Akt pathways in the presence of HGF and EGFR ligands requires inhibition of both EGFR and c-Met. These signaling endpoints demonstrate that downstream biological effects of c-Met and EGFR pathways are redundant and provide rationale for a dual targeting strategy. Consistent with these results, a recent proteomic analysis also revealed extensive overlap between the HGF and EGF signaling networks in lung cancer cells [18]. 

Circulating HGF has previously been reported to be elevated in serum from patients with other cancers including head and neck squamous cell, breast, colorectal and gastric cancers [19]. Recently, NSCLC patients with high serum HGF concentrations who were treated with an EGFR TKI were shown to have a shorter progression-free survival (PFS) and overall survival (OS) in both EGFR wild-type and mutant patients [20]. High serum AREG and/or TGFα levels have been shown to be a predictor of poor response to gefitinib in NSCLC patients with no association between EGFR mutation status and serum AREG and TGFα levels [21]. Our results suggest that later stage NSCLC may have the highest HGF serum levels, while this is not the case for serum AREG or TGFα. The molecular mechanism of AREG overexpression and resistance to gefitinib has recently been described as involving a decrease in gefitinib-mediated apoptosis due to a decrease and sequestering of the proapoptotic BAX protein in the cytoplasm [22]. In contrast, a recent examination of EGFR TKI efficacy in only EGFR wild-type NSCLC patients reported that patients with positive AREG expression in the tumors by immunohistochemistry had a significantly longer PFS and OS [23]. It is clear that much work is still needed to understand the relationship between circulating ligands in patient serum, tumor expression of ligands and receptors as well as EGFR mutation status. Since NSCLC patients without EGFR mutations also have some benefit to EGFR inhibitor therapy, it will be important to explore this combination of inhibitors in both EGFR mutant and wild-type patients. 

We observed that combined inhibition of both the EGFR and c-Met pathways resulted in optimal inhibition of carcinogen-induced tumorigenesis in our novel human HGF overexpressing animal model. Number of tumors per animal as well as downstream signaling and proliferation was maximally inhibited in the dual therapy group compared to single agent treatment and controls. Combined targeting of these two pathways using a variety of different inhibitors has shown benefit in other model systems as well. For example, maximal decreases in cell proliferation and invasion of malignant mesothelioma cells *in vitro* was observed by targeting c-Met and EGFR together [24]. In head and neck squamous cell carcinoma, a synergistic effect on cell growth was observed when combining a c-Met TKI with erlotinib [25]. L2G7 had increased effects in combination with erlotinib in an EGFR mutant glioblastoma model [26]. Another c-Met inhibitor, SGX523, also had better anti-tumor effects when combined with erlotinib in an HGF overexpressing scid mouse model [27]. A phase II clinical trial for advanced NSCLC patients comparing c-Met inhibitor ARQ197 in combination with erlotinib to placebo plus erlotinib demonstrated a significant increase in median PFS of 16.1 weeks for the ARQ197/erlotinib group compared to 9.7 weeks for the placebo/erlotinib group [28]. This survival benefit was most prominent in patients whose tumors were non-squamous histology, K-*ras* mutant and EGFR wild-type.

K-*ras* is one of the most commonly mutated oncogenes in lung cancer and has recently been identified as a biomarker for lack of response to certain anti-cancer therapies [29]. There is a need to determine how K-*ras* mutation affects sensitivity to targeted therapies. Our data suggest that the HGF transgene promotes both K-*ras* mutant and wild-type tumors, but K-*ras* mutant tumors are less responsive to HGF signaling withdrawal by a neutralizing antibody; this was not observed for EGFR signaling inhibition or for inhibition with c-Met TKI PF2341066. A recent report suggested mutant K-*ras* requires interaction with phosphorylated c-Met for maximal signaling to NFκB [30]. Ligand-independent c-Met phosphorylation, which is activated by EGFR in NSCLC [10], could induce interaction with mutant K-*ras*, and this would not be inhibited by a HGF NA, a possible reason for resistance. However, lung tumors in this model with K-*ras* mutation appear still responsive to gefitinib, which would inhibit c-Met transactivation by EGFR in addition to inhibiting EGFR itself [9]. The c-Met TKI did not demonstrate the same resistance to K-*ras* mutation as did an HGF NA. This suggests that blocking the tyrosine kinase action of c-Met, which will inhibit both c-Met ligand-dependent and ligand-independent signaling, is more effective against K-*ras* mutants than blocking ligand-dependent signaling alone.

In summary, circulating ligands for the c-Met and EGFR pathways are both elevated in many lung cancer patients, and signaling from these ligands is compensatory. Targeting both pathways using either HGF neutralizing antibody L2G7 or c-Met TKI PF2341066 with gefitinib yielded maximum anti-tumorigenic effects in the HGF TG model. L2G7 has now been humanized (TAK-701) and is in phase I trials. PF2341066 is also in phase II/III clinical trials. The cross-talk and balance between the c-Met and EGFR intersecting pathways may be critical to response to these therapies. Together these findings provide strong mechanistic rationale for the clinical use of inhibitors of the c-Met pathway in combination with EGFR TKIs in treatment of lung cancer patients and may have implications in other cancers that demonstrate c-Met-EGFR cross-talk as well.

## Correction

Correction Correction (PDF, 17 KB)
