# Supplementary material for: Targeting of Both the c-Met and EGFR Pathways Results in Additive Inhibition of Lung Tumorigenesis in Transgenic Mice
Source: Cancers (Basel). 2010 Dec 22;2(4):2153–70. doi: 10.3390/cancers2042153 (PMC3049550; doi:10.3390/cancers2042153)
Supplement: Correction — (PDF, 17 KB) [file cancers-02-02153-s001.pdf]

Cancers **2010**, *2*

OPEN ACCESS

cancers

ISSN 2072-6694
www.mdpi.com/journal/cancers

Correction

Stabile et al. Targeting of Both the c-Met and EGFR Pathways Results in Additive Inhibition of Lung Tumorigenesis in Transgenic Mice

Laura P. Stabile [1,6], Mary E. Rothstein [1], Phouthone Keohavong [2,6], Diana Lenzner [3], Stephanie R. Land [3,6], Autumn L. Gaither-Davis [1,6], K. Jin Kim [4], Naftali Kaminski [5,6] and Jill M. Siegfried [1,6,*]

1. Department of Pharmacology and Chemical Biology, University of Pittsburgh, Pittsburgh, PA 15213, USA; E-Mails: las22@pitt.edu (L.P.S.); meb63@pitt.edu (M.E.R.); gaitherdavisa@upmc.edu (A.L.G.D.)
2. Department of Environmental and Occupational Health, University of Pittsburgh, Pittsburgh, PA 15213, USA; E-Mail: pho1@pitt.edu
3. Department of Biostatistics, University of Pittsburgh, Pittsburgh, PA 15213, USA; E-Mails: del9@pitt.edu (D.L.); Land@nsabp.pitt.edu (S.R.L.)
4. Galaxy Biotech, LLC, Sunnyvale, CA 94089, USA; E-Mail: jin.kim@galaxybiotech.com
5. Department of Medicine, University of Pittsburgh, Pittsburgh, PA 15213, USA; E-Mail: nak38@pitt.edu
6. Lung and Thoracic Malignancy Program, University of Pittsburgh, Pittsburgh, PA 15213, USA

* Author to whom correspondence should be addressed; E-Mail: siegfriedjm@upmc.edu; Tel.: +1-412-623-7769; Fax: +1-412-623-7768.

Received: *17 January 2011/ Accepted: 17 January 2011 / Published: 17 January 2011*

We have found a mistake in our paper recently published in Cancers [1]. The funding agency and number of a grant that supported the work of one of the co-investigators that provided a part of the data published in this manuscript was not present in the Acknowledgment. The correct Acknowledgment is provided below:

This work was supported by NIH R01 CA79882, SPORE P50CA9045440, NIH/NCI R21 CA129260 and UPCI Cancer Center Support Grant P30 CA047904 for shared facility use. We thank Lisa Chedwick in Research Histological Services at the University of Pittsburgh for technical assistance with the immunohistochemistry experiments and Joel Weissfeld for providing demographic data for control samples used in this study.

Reference

1. Stabile, LP; Rothstein, ME; Keohavong, P; Lenzner, D; Land, SR; Gaither-Davis, AL; Kim, KJ; Kaminski, N; Siegfried, JM. Targeting of Both the c-Met and EGFR Pathways Results in Additive Inhibition of Lung Tumorigenesis in Transgenic Mice. *Cancers* **2010**, 2, 2153-2170.

© 2011 by the authors; licensee MDPI, Basel, Switzerland. This article is an open access article distributed under the terms and conditions of the Creative Commons Attribution license (http://creativecommons.org/licenses/by/3.0/).
